# Elevated Serum Levels of Alpha-Fetoprotein in Patients with Infantile Hemangioma Are Not Derived from within the Tumor

**DOI:** 10.3389/fsurg.2016.00005

**Published:** 2016-02-09

**Authors:** Tinte Itinteang, Alice M. Chibnall, Reginald Marsh, Jonathan C. Dunne, Sophie de Jong, Paul F. Davis, Philip Leadbitter, Swee T. Tan

**Affiliations:** ^1^Gillies McIndoe Research Institute, Wellington, New Zealand; ^2^University of Auckland, Auckland, New Zealand; ^3^Centre for Biodiscovery, School of Biological Sciences, Victoria University of Wellington, Wellington, New Zealand; ^4^Centre for the Study and Treatment of Vascular Birthmarks, Wellington Regional Plastic, Maxillofacial and Burns Unit, Wellington, New Zealand; ^5^Department of Paediatrics, Hutt Hospital, Wellington, New Zealand

**Keywords:** alpha-fetoprotein, infantile hemangioma, endoderm, primitive mesoderm, extrahepatic

## Abstract

**Aims:**

The embryonic-like stem cell origin of infantile hemangioma (IH) and the observed elevated serum levels of alpha-fetoprotein (AFP) in patients with hepatic IH led us to investigate if this tumor was the source of AFP.

**Materials and methods:**

We measured serial serum levels of AFP in patients with problematic proliferating IH treated with surgical excision or propranolol treatment. We also investigated the expression of AFP in extrahepatic IH samples using immunohistochemical staining, mass spectrometry, NanoString gene expression analysis, and *in situ* hybridization.

**Results:**

Serum levels of AFP normalized following surgical excision or propranolol treatment. Multiple regression analysis for curve fittings revealed a different curve compared to reported normal values in the general populations. AFP was not detected in any of the IH samples examined at either the transcriptional or translational levels.

**Conclusion:**

This study demonstrates the association of proliferating IH with elevated serum levels of AFP, which normalized following surgical excision or propranolol treatment. We have shown that IH is not the direct source of AFP. An interaction between the primitive mesoderm-derived IH and the endogenous endodermal tissues, such as the liver, *via* an intermediary, may explain the elevated serum levels of AFP in infants with extrahepatic IH.

## Introduction

Infantile hemangioma (IH) typically undergoes rapid proliferation during infancy (proliferative phase) followed by spontaneous slow involution over the next 1–5 years (involuting phase) with continued improvement up to 10 years, often leaving a fibro-fatty residuum (involuted phase) (1, 2). Recent studies have demonstrated an abundance of stem cells expressing embryonic stem cell (ESC) markers in IH during the proliferative phase (3–5). IH-derived stem cells possess the capability to produce mesoderm and ectoderm downstream derivatives, including adipocytes, osteoblasts, erythrocytes, and neuronal cells, confirming their primitive nature (4, 6–8).

Of interest, these stem cells in IH that express ESC markers do not form teratomas *in vivo* (3, 4) that are typically observed in ESC-derived tumors (9). This infers that the stem cell population within IH is downstream of ESC, with published data showing this primitive population being localized to the endothelium of the microvessels (3, 5, 10).

Recent investigations into the biology of IH suggest a placental chorionic villous mesenchymal core cell (PCVMCC) origin of IH (11). This is supported by the expression profile of IH showing the primitive mesoderm origin of these primitive cells (10) and the unique co-expression of proteins common to both IH and placenta (11, 12). This would imply that the cells from the primitive streak in the fetus proper that eventually give rise to the PCVMCC also give rise to cells of the yolk sac (13). These primitive cells are presumed to embolize into the fetus proper *in utero via* the umbilical vein, through the portal vein, with the liver being the first fetal transition organ prior to their ultimate entry into the fetal arterial circulation (11). This may account for the increased risk of hepatic involvement in patients with multiple cutaneous IH lesions (14).

Alpha-fetoprotein (AFP), a protein similar to albumin, is normally produced by the liver, yolk sac, and the gastrointestinal tract (15). It is an important biomarker for certain tumors and liver diseases in childhood (16). Interestingly AFP is also associated with the onset of endodermal differentiation from ESC (17). Elevated serum levels of AFP have also been reported in patients with hepatic IH (18) and infantile hepatic hemangioendothelioma (19, 20).

Previous reports documenting increased levels of circulating AFP in patients with hepatic IH led to the hypothesis that IH may be the source of circulating AFP, rather than being the effector as previously proposed (19). However, what remain to be determined are the serum levels of AFP in infants with extrahepatic IH, compared to the physiologically elevated levels postnatally.

This study analyzed serial serum levels of AFP in patients undergoing surgical excision or propranolol treatment for problematic extrahepatic IH. We also investigated the expression of AFP in IH tissues at the transcriptional and translational levels to determine if IH was the source of AFP production.

## Materials and Methods

### Analysis of Serum Levels of AFP

Patients with problematic proliferating IH aged 2–12 (mean, 5.8) months were prospectively recruited from our Vascular Anomalies Center, in a study approved by the Central Health and Disability Ethics Committee. The demographic data of the patients and the characteristics of their IH are presented in Table 1. Patients treated conservatively were excluded from the study.

**Table 1:** **Demographic details of the patients and characteristics of their problematic proliferating infantile hemangioma**.

Patient	Treatment	Age (months)	Size of main lesion (cm)	Site of main lesion	Number of lesions	Sex	Gestation (weeks)
1	Propranolol	5	10 × 8	Chest	1	F	36
2	Propranolol	11	4 × 3.5	Shoulder	1	F	41
3	Propranolol	3	10 × 8	Cheek/parotid	1	F	40
4	Propranolol	3	0.8 × 0.8	Upper lip	1	F	34
5	Propranolol	7	1 × 1	Lower eyelid	1	F	38
6	Propranolol	3	1.5 × 1.5	Nose	4	M	32
7	Propranolol	3	12 × 4	Elbow	1	F	40
8	Propranolol	3	2 × 3	Ear	1	F	36
9	Propranolol	2	2.5 × 5	Nose	1	M	39
10	Propranolol	3	8 × 5	Arm	1	F	39
11	Propranolol	5	2 × 1.5	Upper eyelid	1	F	40
12	Propranolol	4	8 × 8	Buttock	1	M	40
13	Surgery	7	2 × 2	Neck	1	M	33
14	Surgery	6	4 × 3	Buttock/back	2	M	35
15	Surgery	7	2.5 × 3.5	Labia majora	1	F	40
16	Surgery	8	2 × 2	Mons pubis	1	F	39
17	Surgery	12	3 × 3	Scalp	1	F	38
18	Surgery	4	2.5 × 2	Natal cleft	1	M	40
19	Surgery	9	2 × 2	Scalp	1	F	38
20	Surgery	5	3.5 × 3	Labia	1	F	40
21	Surgery	12	1.5 × 1.5	Eyebrow	2	F	40
22	Surgery	7	2.5 × 1.5	Temple	2	F	37
23	Surgery	6	2.5 × 2.5	Neck	2	F	41
24	Surgery	4	3 × 1.5	Scalp	1	M	40

*M, male; F, female*.

All patients underwent blood collection by venipuncture before the initiation of propranolol treatment. For patients undergoing surgical excision, blood samples were obtained immediately following induction of anesthesia. Blood samples were collected 3, 6, and 9 months following surgical excision or initiation of propranolol treatment. Blood samples were analyzed for AFP at the Capital and Coast Laboratory (Wellington, NZ) using Roche Cobas e601 by Electrochemiluminescence (reference range, 0–10 kU/L) and were converted into nanograms per milliliter for each measurement, to be consistent with the value in published reports (21, 22). Participants with elevated levels of AFP, based on normal values reported in the literature (21, 22) underwent liver ultrasonography to exclude hepatic pathology.

### Tissue Samples

Surgically excised proliferating (*n* = 6, including three from the cohort of patients in this study) and involuted (*n* = 6) IH tissues obtained from patients aged 3–10 (mean, 6) months and 6–12 (mean 9) years, respectively, were used for immunohistochemical (IHC) and mRNA *in situ* hybridization (ISH) staining, NanoString, and mass spectrometry analyses.

### Immunohistochemical Staining

4-μm-thick formalin-fixed paraffin-embedded IH sections underwent 3,3-diaminobenzidine (DAB) immunohistochemical IHC staining with the primary antibodies for AFP (ready-to-use, Leica, Sydney, Australia) and smooth muscle actin (SMA, ready-to-use, Leica) on the Bond Rx Autostainer (Leica) using an established protocol (23–25). All slides were counterstained with hematoxylin (Leica) and mounted with Surgipath Micromount Media (Leica). Infant liver sections were used as a positive control for AFP.

### *In Situ* Hybridization

Proliferating (*n* = 3) and involuting (*n* = 3) IH samples from the same cohorts used for IHC staining were subjected to ISH using the AFP probe (NM_001134, Affymetrix, CA, USA) on the Bond Rx Autostainer (Leica) using a recently described protocol (23). Infant liver sections were used as a positive control. All slides were counterstained with hematoxylin (Leica).

### NanoString Gene Expression Analysis

The RNeasy Mini Kit (Qiagen) was used on snap-frozen proliferating (*n* = 6) and involuted (*n* = 6) IH samples to extract RNA. The RNA samples were validated using the NanoDrop2000 Spectrophotometer (Thermo Scientific). The samples were then analyzed with the NanoString nCounter™ Gene Expression Assay (NanoString Technologies, Seattle, WA, USA), which was conducted by New Zealand Genomics Ltd. (Dunedin, NZ, USA) according to the manufacturer’s protocol. NanoString Technologies designed and synthesized the probes for the genes encoding AFP (NM_000789.2) and the house keeping gene GAPDH (NM_002046.3). The standard settings of nSolver software (NanoString Technologies) were used to normalize and validate the data against in house controls. The data were expressed as a ratio over the housekeeping gene for normalization.

### Mass Spectrometry

Total protein extraction, digestion, and mass spectrometry were performed as described previously (24). Briefly, snap-frozen proliferating (*n* = 2) and involuted (*n* = 2) IH tissues from the same cohorts used for IHC staining were homogenized with a Dounce Homogenizer (Thomas Co., Philadelphia, PA, USA) in ice-cold RIPA buffer (Sigma-Aldrich, St. Louis, MA, USA) containing Complete Protease inhibitor (Roche Life Science, Penzberg, Germany), and after quantitation (Qubit^®^ 2.0 Fluorometer, Life Technologies, San Diego, CA, USA), 100 mg of total protein per sample was precipitated overnight at −20°C (ProteoExtract^®^ Protein Precipitation Kit, Merck Millipore, Billerica, MA, USA). Protein pellets were resuspended in 100 mM TEAB buffer (pH 8.5) (Sigma-Aldrich) containing 5% sodium deoxycholate (SDC, Merck, Darmstadt, Germany) and 10 mM dithiothreitol (Sigma-Aldrich) and incubated at 80°C for 30 min. After alkylation (40 mM iodoacteamide, Sigma-Aldrich) and dilution (10-fold in 100 mM TEAB buffer, pH 8.5), digestion was performed overnight at 37°C using 4 mg trypsin (Roche Life Science, Penzberg, Germany) per sample. SDC was removed by formic acid (Merck) precipitation (1% final). After lyophilization to ~10 μL, each sample was reconstituted in 0.1% formic acid, purified (OMIX C18 pipette tip, Agilent Technologies, Santa Clara, CA, USA), and prepared for LC–MS/MS.

LC–MS/MS was performed using an UltiMate 3000 HPLC system (Dionex, Thermo Scientific, Waltham, MA, USA) and LTQ Orbitrap XL mass spectrometer (Thermo Scientific, Waltham, MA, USA) and each sample was analyzed in quadruplicate. Eluted peptides were analyzed using data-dependent MS/MS acquisition, and raw MS/MS data files were searched against a complete human protein database (SwissProt KB, October 22, 2014, 69689 sequences) using Proteome Discoverer™ V1.4 (Thermo Scientific, Waltham, MA, USA) and Scaffold 4.0 (Proteome Software, Portland, OR, USA). Peptide assignments were accepted above 90% confidence, and protein identification parameters were: protein threshold, 1.0% FDR; minimum total spectrum count, 2; and peptide threshold, 1.0% FDR.

### Statistical Analysis of Serum Levels of AFP

To determine the significance of surgical excision or propranolol treatment on serial AFP levels for our IH patients, we performed *t* tests, using SPSS (V.22) for paired samples before and 1 month after surgical excision or propranolol treatment.

Serum levels of AFP recorded by the age (in months) of IH patients were compared with the norms for postnatal levels of AFP in the general American (21) and Japanese (22) populations. Conversion from our original kilounits per liter AFP units to the nanograms per milliliter units of the US (21) and Japanese (22) norms, as expected, left the correlation values unchanged but with the *F* value still massive but halved by rounding effects. As there is no convenient significance test of the difference in *distributions of IH individual’s levels* and *distributions of mean levels* over time, a curve fitting exercise was done to investigate the difference between serial serum levels of AFP in IH patients in this study and the normal levels. SPSS (V.22) curve fit was used for 11 different types of distributions: linear, growth, quadratic, compound, logarithmic, cubic, *S*, exponential, inverse, power, and logistic.

## Results

### Analysis of Serum Levels of AFP

Of the 24 patients recruited into this study, 12 underwent surgical excision and 12 had propranolol treatment. Table 1 shows the demographics of the patients and the characteristics of their IH lesions. Liver ultrasonography of the patients with elevated serum AFP revealed no hepatic lesions.

Analysis of serial serum levels of AFP for both the surgical excision and the propranolol treatment cohorts demonstrated tapering serum levels of AFP in both groups, concordant with the patients’ age. The *t* tests comparing pretreatment AFP levels and 1 month posttreatment levels revealed a significant reduction of AFP levels for patients who were on propranolol (*p* = 0.014), but not those who had undergone surgical excision (*p* = 0.189).

**Figure 1:**
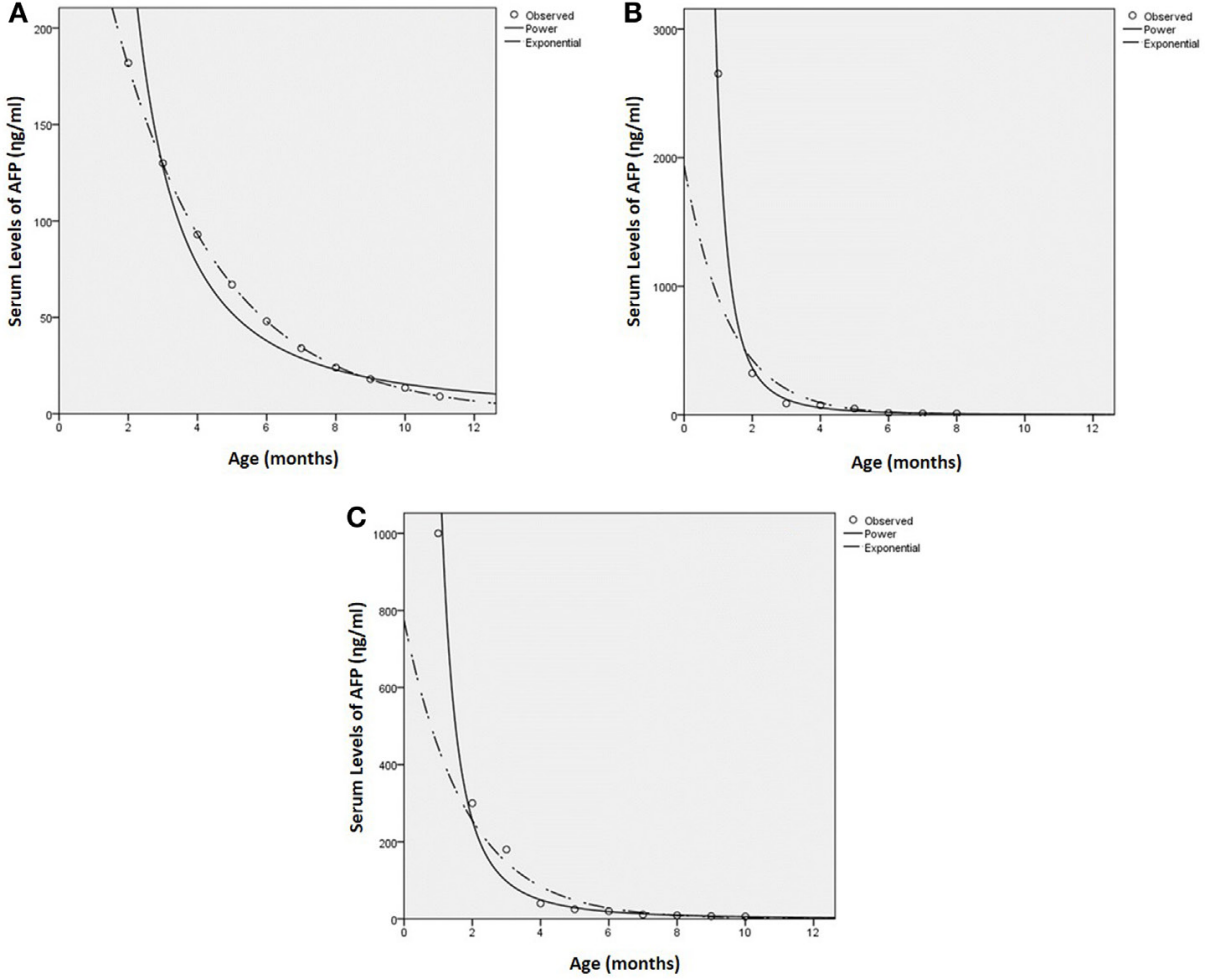
**Curve function for serum levels of AFP for infantile hemangioma (A), normal American** (21) **(B), and Japanese** (22) **(C) children, with the analysis values for each of the groups**.

Analysis of the serial AFP levels based on the results of previously reported normal serum levels of AFP in the American (Figure 1B) (21) and Japanese (Figure 1C) (22) populations are effectively the same with *R*^2^’s of 0.978 and 0.979 respectively, and *F* values of 371.5 and 271.6 (Table 2), respectively. This leaves the power function with the better fit for the norms than the exponential function with respective R2‘s of 0.910 and 0.901 and associated *F* values of 271.6 and 80.6. It is in contrast to the group of IH patients in our study, which gave a power function fit of *R*^2^ = 0.948 with an *F* of 145.6, but an exponential function fit of *R*^2^ = 0.999 (Table 2) with an *F* of 15028.5, giving a perfect fit for the exponential function (Figure 1A). Thus Figures 1A–C illustrate the plots of the individual datasets with the functional analysis, demonstrating that the pattern of serial serum levels of AFP in children with IH are substantially different from those of the norms for normal American (21) and Japanese (22) children.

**Table 2:** **Functional analysis of serial serum levels of AFP in children with infantile hemangioma and normal American (21) and Japanese (22) children**.

	IH		American		Japanese	
Function	*R*^2^	*F*	*R*^2^	*F*	*R*^2^	*F*
Power	0.948	145.5	0.979	371.5	0.978	271.6
Exponential	0.999	7442	0.910	54.6	0.901	80.6

*IH, infantile hemangioma*.

### Immunohistochemical Staining

**Figure 2:**
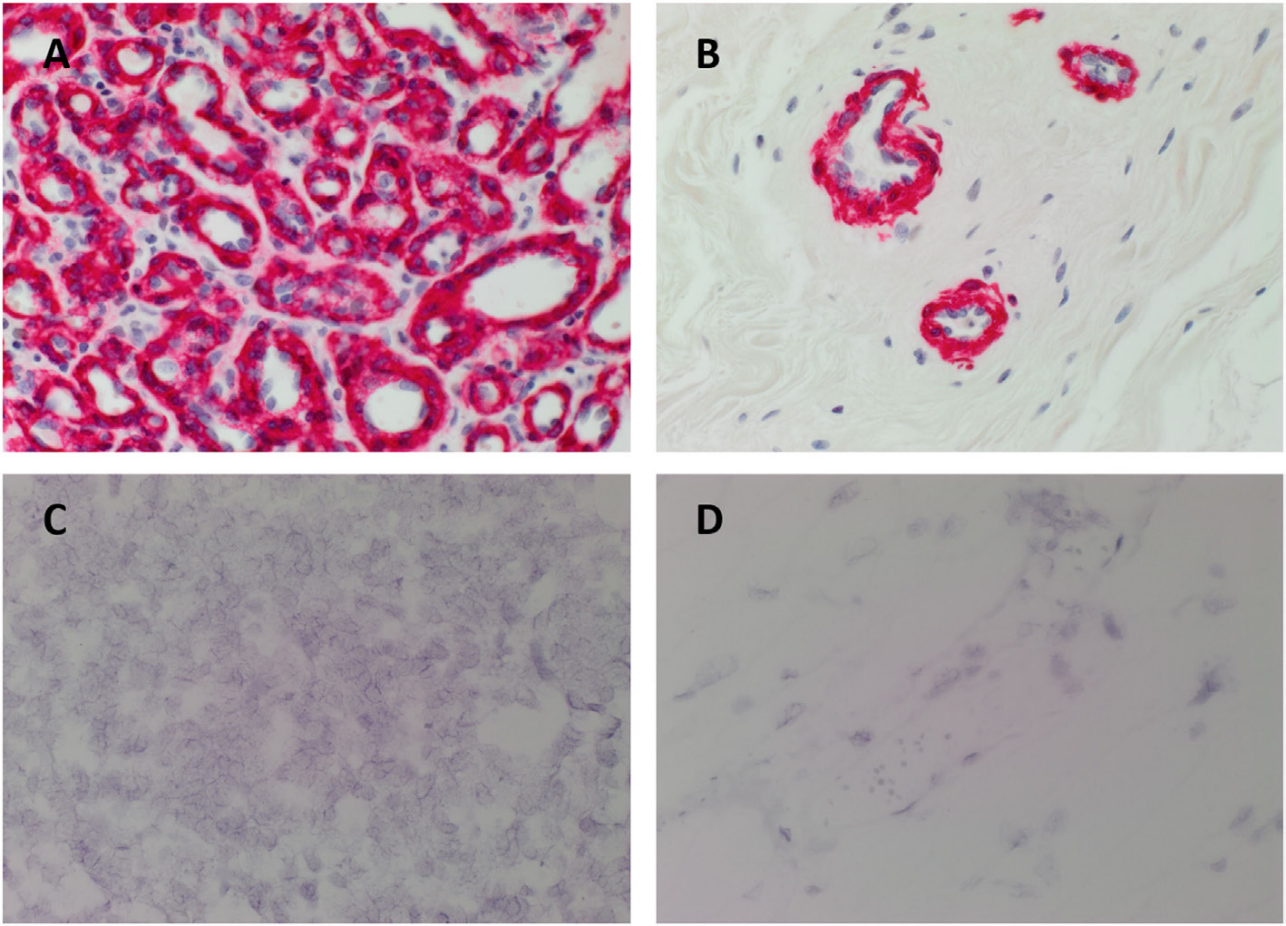
**Representative 3,3-diaminobenzidine immunohistochemical (A,B) and *in situ* hybridization (C,D) stained sections of proliferating (A,C) and involuted (B,D) infantile hemangiomas (IH) demonstrating the absence of AFP [(A,B), brown; (C,D), red]**. The IHC-stained IH sections were also stained with SMA [**(A,B)**, red], identifying the pericyte layer of the microvessels. All slides were counterstained with hematoxylin to identify cell nuclei [**(A–D)**, blue]. Image magnification: 400×.

All IH samples used in this study showed expression of GLUT-1 by the endothelium of the microvessels (data not shown), confirming the diagnosis. To investigate if the increased levels of circulating AFP in patients with proliferating IH were derived from within the IH, we performed IHC staining for AFP in proliferating (Figure 2A, brown) and involuted (Figure 2B, brown) IH lesions. All IH lesions were counterstained with SMA (Figures 2A,B, red) to demonstrate the pericyte layer of the microvessels. There was no expression of AFP in all the lesions examined. The normal infant liver sections used as a positive control demonstrated positive staining (Image S1A in Supplementary Material, brown).

### *In Situ* Hybridization

To confirm the absence of AFP in IH with IHC staining we investigated for the presence of AFP at the transcriptional level. All IH samples used for ISH did not show positive staining for AFP in both proliferating (Figure 2C, red) and involuted (Figure 2D, red) samples. Specificity of the AFP probe was confirmed with infant liver sections as a positive control (Image S1B in Supplementary Material, red).

### NanoString Gene Expression Analysis

To validate the results of ISH staining, copy number of AFP mRNA was analyzed in the same proliferating (*n* = 6) and involuted (*n* = 6) IH lesions from the cohorts used for IHC staining. The mRNA expression for AFP was not detectable above the levels of the negative controls (data not shown).

### Mass Spectrometry

Total protein extracts from proliferating (*n* = 2) and involuted (*n* = 2) IH tissue samples from the same cohorts used for IHC staining were trypsin digested and analyzed by liquid chromatography–orbitrap mass spectrometry. Searching against a complete human protein database resulted in the identification of 773 proteins with a minimum of two matched tryptic peptides (1.2% FDR), of which 682 were common to both phases of IH. Despite allowing for up to two missed tryptic cleavage sites, which generated 157 theoretical tryptic peptides from the full-length protein, AFP was not detected in any of the IH samples analyzed (data not shown).

## Discussion

Recent advances in the understanding of IH have revealed a novel stem cell *niche* (1, 4, 5, 10, 26) with a putative PCVMCC origin (11). These placenta-derived cells share a common origin with the primitive mesoderm cells of the yolk sac, which is the source of AFP *in utero* (27). Normalization of serum levels of AFP following surgical excision or propranolol treatment supports the association of circulating AFP and IH, as described in this report. The contrasting exponential decay patterns of serial serum levels of AFP in IH infants following surgical excision or propranolol treatment reported in this study, compared with the normal populations (21, 22), further supports an association between IH and increased serum levels of AFP. However, what remains unknown is whether IH is directly or indirectly responsible for the increased AFP production.

Elevated serum levels of AFP have been reported in patients with hepatic IH (18). Elevated serum levels of AFP have also been reported in infants with hepatic hemangioendothelioma (19, 20), with AFP being expressed on the surrounding hepatocytes (28).

A previous publication reports the absence of AFP in IH, based solely on IHC staining (29). To the best of our knowledge, this is the first report showing the absence of the expression of AFP in extrahepatic proliferating IH lesions at both the transcriptional and translational levels.

Physiologically serum levels of AFP are very high at birth, and taper exponentially postnatally to near normal adult levels (20 ng/mL) by 8–9 months of age (15, 30). However, given the association of AFP production with hepatic tumors, such as hepatocellular carcinoma and hepatoblastoma (31), we have performed liver ultrasonography in infants with high levels of AFP in this study to exclude the presence of these tumors. Interestingly, none of the IH patients with high serum levels of AFP in this study showed hepatic pathology. This suggests an association between IH and raised serum levels of AFP, which is independent of liver involvement by IH.

Recent demonstration of the expression of ESC markers in proliferating IH, coupled with the speculated capability of IH-derived cells to undergo *in vitro* differentiation down an endodermal lineage that expresses AFP, led us to hypothesize that IH may be the direct source of AFP production. Intriguingly, we were not able to detect the expression of AFP in any of the IH samples examined in this study, at either the transcriptional or translational level. We speculate that the elevated serum levels of AFP are indirectly caused by IH, possibly through one or more intermediaries between the IH and downstream AFP production.

The endodermal element of the yolk sac that produces AFP *in utero* (27) is situated immediately adjacent to the outer extra-embryonic mesoderm, which is derived from the primitive mesoderm (13), the putative origin of IH (10, 11). It is exciting to speculate that an interaction between the primitive mesoderm derived IH, and the endogenous endodermal tissues, such as liver, results in AFP production by the latter. This suggests possible secretion of a messenger protein by proliferating IH that causes a tissue of endodermal origin to produce AFP. However, this is the topic of further investigation.

This study is the first to demonstrate elevated serum levels of AFP in patients with non-hepatic proliferating IH and the absence of AFP in IH tissue samples. An interaction between the primitive mesoderm derived IH, and the endogenous endodermal tissues, such as liver, *via* an intermediary, may explain the elevated serum levels of AFP in infants with extrahepatic IH.

## Ethics

This study was approved by the Central Health and Disability Ethics Committee (reference no: CEN/12/06/023).

## Author Contributions

TI, PFD, and STT formulated the hypothesis of the research and designed the study. SdJ, PL, and STT provided the clinical and serum data. TI performed the IHC and ISH experiments and analysis of the data. RM performed the serum AFP statistical analysis. AC analyzed the NanoString data. JCD performed the mass spectrometry experiments and analysis of the data. TI and STT drafted the manuscript. All authors read and approved the manuscript.

## Conflict of Interest Statement

The authors declare that the research was conducted in the absence of any commercial or financial relationships that could be construed as a potential conflict of interest.

## References

[1] Itinteang T Withers AHJ Davis PF Tan ST. Biology of infantile hemangioma. Front Surg (2014) 1:38. 10.3389/fsurg.2014.00038 25593962 PMC4286974

[2] Munden A Butschek R Tom W Marshall J Poeltler D Krohne S Prospective study of infantile haemangiomas: incidence, clinical characteristics and association with placental anomalies. Br J Dermatol (2014) 170:907–13. 10.1111/bjd.12804 24641194 PMC4410180

[3] Itinteang T Tan ST Brasch HD Steel R Best HA Vishvanath A Infantile haemangioma expresses embryonic stem cell markers. J Clin Pathol (2012) 65:394–8. 10.1136/jclinpath-2011-200462 22447921

[4] Khan Z Boscolo E Picard A Psutka S Melero-Martin J Bartch T Multipotential stem cells recapitulate human infantile hemangioma in immunodeficient mice. J Clin Invest (2008) 118:2592–9. 10.1172/JCI33493 18535669 PMC2413184

[5] Huang L Nakayama H Klagsbrun M Mulliken J Bischoff J. Glucose transporter 1-positive endothelial cells in infantile hemangioma exhibit features of facultative stem cells. Stem Cells (2015) 33:133–45. 10.1002/stem.1841 25187207 PMC4270824

[6] Itinteang T Vishvanath A Day DJ Tan ST. Mesenchymal stem cells in infantile haemangioma. J Clin Pathol (2011) 64:232–6. 10.1136/jcp.2010.085209 21242328

[7] Yu Y Fuhr J Boye E Gyorffy S Soker S Atalia A Mesenchymal stem cells and adipogenesis in hemangioma involution. Stem Cells (2006) 24:1605–12. 10.1634/stemcells.2005-0298 16456130

[8] Itinteang T Tan ST Brasch HD Vishvanath A Day DJ. Primitive erythropoiesis in infantile haemangioma. Br J Dermatol (2011) 164:1097–100. 10.1111/j.1365-2133.2010.10187.x 21518328

[9] Hentze H Soong P Wang S Phillips B Putti T Dunn N. Teratoma formation by human embryonic stem cells: evaluation of essential parameters for future safety studies. Stem Cell Res (2009) 2:198–210. 10.1016/j.scr.2009.02.002 19393593

[10] Itinteang T Tan ST Brasch HD Day DJ. Primitive mesodermal cells with a neural crest stem cell phenotype predominate proliferating infantile haemangioma. J Clin Pathol (2010) 63:771–6. 10.1136/jcp.2010.079368 20696686

[11] Itinteang T Tan ST Guthrie S Tan CES McIntyre B Brasch HD A placental chorionic villous mesenchymal core cellular origin for infantile haemangioma. J Clin Pathol (2011) 64:870–4. 10.1136/jclinpath-2011-200191 21947300

[12] North P Warner M Mizeracki A Mrak RE Nicholas R Kincannon J A unique microvascular phenotype shared by juvenile hemangiomas and human placenta. Arch Dermatol (2001) 137:559–70. 10.1001/archderm.137.12.1607 11346333

[13] Kinder S Tsang TE Quinlan GA Hadjantonakis A Nagy A Tam PP. The orderly allocation of mesodermal cells to the extraembryonic structures and the anteroposterior axis during gastrulation of the mouse embryo. Development (1999) 126:4691–701. 10518487 10.1242/dev.126.21.4691

[14] Horri K Drolet A Baselga E Frieden I Metry D Morel K Risk of hepatic hemangiomas ininfants with large hemangiomas. Arch Dermatol (2010) 146:201–3. 10.1001/archdermatol.2009.391 20157038

[15] Wu J. Serum alpha-fetoprotein and its lectin reactivity in liver diseases: a review. Ann Clin Lab Sci (1990) 20:98–105. 1691611

[16] Bader D Riskin A Vafsi O Tamir A Peskin B Israel N Alpha-fetoprotein in the early neonatal period – a large study and review of the literature. Clin Chim Acta (2004) 349:15–23. 10.1016/j.cccn.2004.06.020 15469851

[17] Abe K Niwa H Takiguchi M Mori M Abe K Yamamura KT Endoderm-sepcific gene expression in embryonic stem cells differentiated to embryoid bodies. Exp Cell Res (1996) 229:27–34. 10.1006/excr.1996.0340 8940246

[18] Kapoor G Kurkure P Borwankar S Advani S Interpretation of serum alpha-fetoprotein in an infant with hepatomegaly. Indian Pediatr (1996) 33:65–9. 8772959

[19] Kim E Koh K Park M Kim H Seo J Clinical features of infantile hepatic hemangioendothelioma. Korean J Pediatr (2011) 54:260–6. 10.3345/kjp.2011.54.6.260 21949521 PMC3174362

[20] Zenge J Fenton L Lovell M Grover T. Case report: infantile hemangioendothelioma. Curr Opin Pediatr (2002) 14:99–102. 10.1097/00008480-200202000-00018 11880743

[21] Wu J Brook L Sudar K. Serum alpha fetoprotein (AFP) levels in normal infants. Pediatr Res (1981) 15:50–2. 10.1203/00006450-198101000-00012 6163129

[22] Ohama K Nagase H Ogino K Tsuchida K Tanaka M Kubo M. Alpha-fetoprotein (AFP) levels in normal children. Eur J Pediatr Surg (1997) 7:267–9. 10.1055/s-2008-1071168 9402482

[23] Tan EMS Itinteang T Chudakova D Marsh R Brasch HD Davis PF Characterisation of lymphocyte sub-populations in infantile haemangioma. J Clin Pathol (2015) 68:812–8. 10.1136/jclinpath-2014-202846 26067666

[24] Itinteang T Chudakova D Dunne J Davis PF Tan ST Expression of cathepsins B, D, and G in infantile haemangioma. Front Surg (2015) 2:26 10.3389/fsurg.2015.00026 26137466 PMC4470331

[25] Tan EMS Chudakova D Davis PF Brasch HD Itinteang T Tan ST. Characterisation of subpopulations of myeloid cells in infantile haemangioma. J Clin Pathol (2015) 68:571–4. 10.1136/jclinpath-2014-202846 25834091

[26] Spock C Tom L Canadas K Sue G Sawh-Martinez R Maier C Infantile hemangiomas exhibit neural crest and pericyte markers. Ann Plast Surg (2015) 74:230–6. 10.1097/SAP.0000000000000080 24401806

[27] Jones E Clement-Jones M James O Wilson D. Differences between human and mouse alpha-fetoprotein expression during early development. J Anat (2001) 198:555–9. 10.1046/j.1469-7580.2001.19850555.x 11430694 PMC1468244

[28] Kim T Lee Y Song Y Park C Shim S Kang C Infantile hemangioendothelioma with elevated serum α fetoprotein: report of 2 cases with immunohistochemical analysis. Hum Pathol (2010) 41:763–7. 10.1016/j.humpath.2009.05.019 20153513

[29] Seo S Min K Mirkin L. Hepatic hemangioendothelioma of infancy associated with elevated alpha fetoprotein and catecholamine by-products. Pediatr Pathol (1988) 8:625–31. 10.3109/15513818809022319 2469076

[30] Bellini C Bonacci W Parodi E Serra G Serum α-fetoprotein in newborns. Clin Chem (1998) 44:2548–50. 9836727

[31] Schneider D Calaminus G Gobel U. Diagnostic value of alpha1-fetoprotein and beta-human chorionic gonadotropin in infancy and childhood. Pediatr Hematol Oncol (2001) 18:11–26. 10.1080/088800101750059828 11205836

