# Supplementary figures and images for: Elevated Serum Levels of Alpha-Fetoprotein in Patients with Infantile Hemangioma Are Not Derived from within the Tumor
Source: Front Surg. 2016 Feb 9;3:5. doi: 10.3389/fsurg.2016.00005 (PMC4746268; doi:10.3389/fsurg.2016.00005)

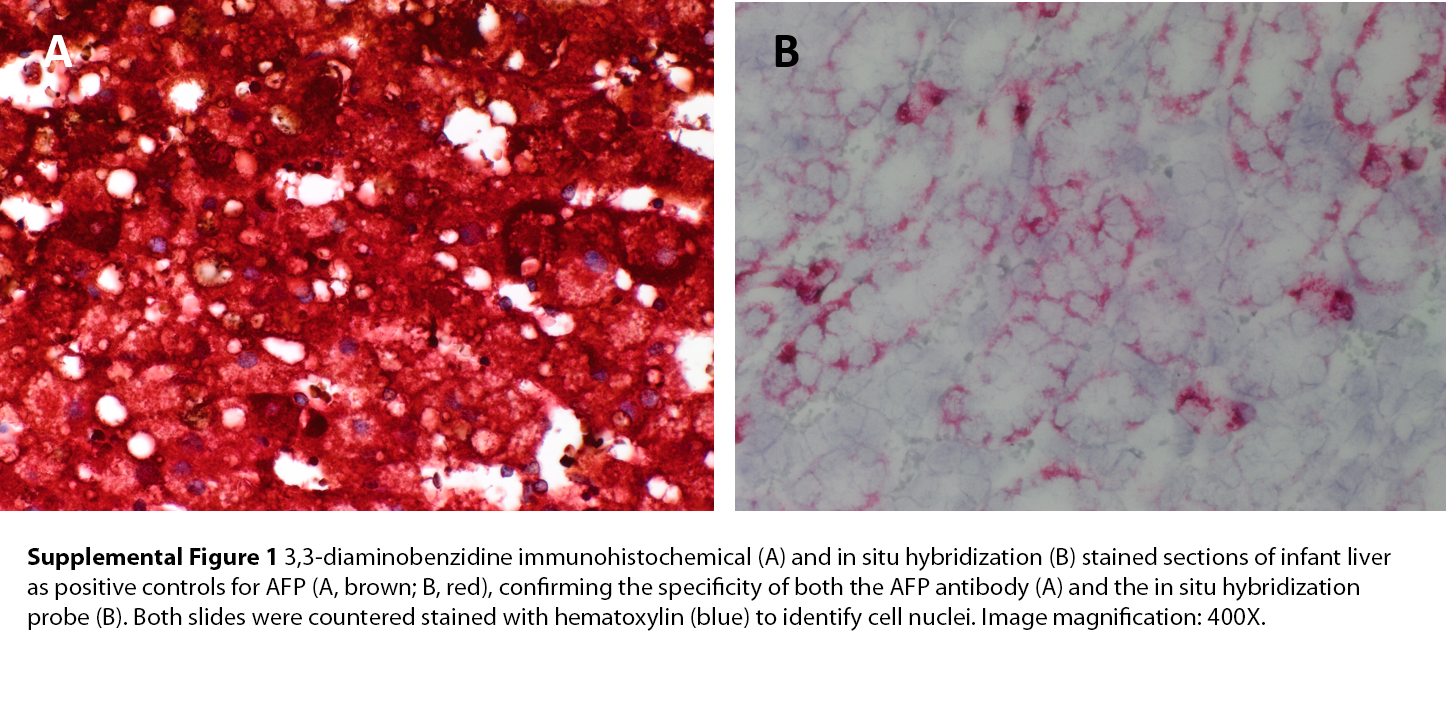

Supplement: Supplementary file 1 [file image_1.tif]
